# The relationship between glomerular IgG staining and poor prognostic findings in patients with IgA nephropathy: the data from TSN-GOLD working group

**DOI:** 10.1186/s12882-021-02560-2

**Published:** 2021-10-28

**Authors:** Kenan Turgutalp, Egemen Cebeci, Aydin Turkmen, Ulver Derici, Nurhan Seyahi, Necmi Eren, Fatih Dede, Mustafa Gullulu, Taner Basturk, Gulizar Manga Sahin, Murvet Yilmaz, Savas Sipahi, Garip Sahin, Sena Ulu, Erhan Tatar, Ali Gundogdu, Rumeyza Turan Kazancioglu, Can Sevinc, Ozkan Gungor, İdris Sahin, Sim Kutlay, Ilhan Kurultak, Zeki Aydin, Bulent Altun, Belda Dursun, Zulfikar Yilmaz, Ozcan Uzun, Gultekin Suleymanlar, Ferhan Candan, Siren Sezer, Derya Basak Tanburoglu, Zerrin Bicik Bahcebasi, Dilek Taymez, Esra Akcali, Deren Oygar, Zulal Istemihan, Simge Bardak, Omer Faruk Akcay, Mevlut Tamer Dincer, Erkan Dervisoglu, Ezgi Yenigun, Kultigin Turkmen, Savas Ozturk

**Affiliations:** 1 Division of Nephrology, Department of Internal Medicine, School of Medicine, Mersin University, 33079 Mersin, Turkey; 2 Department of Nephrology, Haseki Training and Research Hospital, Istanbul, Turkey; 3 Department of Nephrology Istanbul University, Istanbul Faculty of Medicine, Istanbul, Turkey; 4 Department of Nephrology, Gazi University Faculty of Medicine, Ankara, Turkey; 5 Department of Nephrology, Istanbul University-Cerrahpasa, Cerrahpasa Faculty of Medicine, Istanbul, Turkey; 6 Department of Nephrology Kocaeli University, Faculty of Medicine, Kocaeli, Turkey; 7 Department of Nephrology, Health Sciences University, Ankara Numune Education and Research Hospital, Ankara, Turkey; 8 Department of Medicine, Uludag University Faculty of Medicine, Bursa, Turkey; 9 Department of Nephrology, Health Sciences University, Sıslı Hamidiye Etfal Training and Research Hospital, Istanbul, Turkey; 10 Department of Nephrology, Istanbul 2. Abdulhamid Han Training and Research Hospital, Istanbul, Turkey; 11 Department of Nephrology, Health Sciences University, Bakirkoy Sadi Konuk Training and Research Hospital, Istanbul, Turkey; 12 Department of Nephrology, Sakarya University Medical Faculty Education and Research Hospital, Istanbul, Turkey; 13 Department of Nephrology, Eskisehir Osmangazi University, Faculty of Medicine, Eskisehir, Turkey; 14 Department of Nephrology, Faculty of Medicine, Afyon Health Sciences University, Afyon, Turkey; 15 Department of Nephrology, Health Sciences University, Izmir Bozyaka Training and Research Hospital, Izmir, Turkey; 16 Department of Nephrology, Erciyes University, FAculty of Medicine, Kayseri, Turkey; 17 Division of Nephrology, Bezmialem Vakif University, Faculty of Medicine, Istanbul, Turkey; 18 Department of Nephrology, Atatürk University, School of Medicine, Erzurum, Turkey; 19 Department of Nephrology, Kahramanmaras Sutcu Imam University, FAculty of Medicine, Kahramanmaraş, Turkey; 20 Department of Nephrology, Inonu University Faculty of Medicine, Malatya, Turkey; 21 Department of Nephrology, Ankara University, School of Medicine, Ankara, Turkey; 22 Department of Nephrology, Trakya University, Faculty of Medicine, Edirne, Turkey; 23 Department of Nephrology, Darica Farabi Training and Research Hospital, Kocaeli, Turkey; 24 Department of Nephrology, Hacettepe University, Faculty of Medicine Ankara, Ankara, Turkey; 25 Department of Nephrology, Pamukkale University, Scool of Medicine, Denizli, Turkey; 26 Department of Nephrology Dicle University, School of Medicine, Diyarbakır, Turkey; 27 Department of Nephrology Dokuz Eylül University, Faculty of Medicine, İzmir, Turkey; 28 Department of Nephrology Akdeniz University, School of Medicine, Antalya, Turkey; 29 Department of Nephrology Cumhuriyet University, School of Medicine, Sivas, Turkey; 30 Department of Nephrology Atilim University, School of Medicine, Medicana International Hospital, Ankara, Turkey; 31 Department of Nephrology Cukurova University, School of Medicine, Adana, Turkey; 32 Department of Nephrology, Kartal Lutfi Kirdar Training and Research Hospital, Istanbul, Turkey; 33 Department of Nephrology, Kocaeli Government Hospital, Kocaeli, Turkey; 34 Department of Nephrology, Lefkosa BND State Hospital, Lefkosa, Cyprus; 35 Department of Internal Medicine, Division of Nephrology, Necmettin Erbakan University, Meram School of Medicine, Konya, Turkey

**Keywords:** IgA nephropathy, Glomerular IgG staining, Renal prognostic factors

## Abstract

**Background:**

Galactose-deficient IgA1 (Gd-IgA1) has an increased tendency to form immunocomplexes with IgG in the serum, contributing to IgAN pathogenesis by accumulating in the glomerular mesangium. Several studies showed that glomerular IgG deposition in IgAN is an important cause of mesangial proliferation and glomerular damage. This study aims to determine the association of the positivity of IgG and the intensity of IgG staining with a poor renal prognosis.

**Methods:**

A total of 943 IgAN patients were included in the study. Glomerular IgG staining negative and positive patients were compared using Oxford classification scores, histopathological evaluations, proteinuria, eGFR, albumin, blood pressures. IgG positive patients were classified as (+), (++), (+++) based on their staining intensity, and the association with the prognostic criteria was also evaluated.

**Results:**

81% (*n* = 764) of the patients were detected as IgG negative, while 19% (*n* = 179) were positive. Age, gender, body mass index, blood pressure, proteinuria, eGFR, uric acid values were similar in IgG positive and negative patients who underwent biopsy (*p* > 0.05). Intensity of glomerular IgG positivity was not found to be associated with diastolic and systolic blood pressure, urea, uric acid, age, eGFR, albumin, proteinuria (p > 0.05 for all, r = − 0.084, r = − 0.102, r = − 0.006, r = 0.062, r = 0.014, r = − 0.044, r = − 0.061, r = − 0.066, r = 0.150, respectively). There was no difference for histopathological findings between IgG (+), IgG (++), IgG (+++) groups (for all, p > 0.05).

**Conclusion:**

Glomerular IgG negativity and positivity detected by routine IFM in IgAN patients is not associated with poor renal prognostic risk factors.

## Background

IgA nephropathy (IgAN) is the most common cause of primary glomerulonephritis throughout the world, including Turkey [1, 2]. Although the prognosis may be difficult to predict, important risk factors for the progressive disease, including demographic, clinical and histopathological findings, have been identified. Male gender, amount of proteinuria, increased systolic and diastolic blood pressures, hyperuricemia, increased body mass index (BMI) and, increased age) have been defined for poor renal prognosis of IgAN [3]. The diagnosis requires a kidney biopsy, which often provides additional prognostic information: mesangial hypercellularity, endocapillary hypercellularity, segmental glomerulosclerosis, tubular atrophy/interstitial fibrosis and presence of crescents correlated with adverse renal outcomes [3].

Renal biopsies of IgAN patients are characterised by mesangial IgA dominant or codominant immunoglobulin deposits that may have co-deposition of IgG. Compared with healthy subjects, there is an increase in the proportion of Galactose-deficient IgA1(Gd-IgA1) in the serum. Gd-IgA1 has an increased tendency to form immunocomplexes with IgG in the serum, contributing to IgAN pathogenesis by accumulating in the glomerular mesangium. Several studies showed that glomerular IgG deposition in IgAN is an important cause of mesangial inflammation, which causes proliferation of the mesangial cells and glomerular damage. Some studies stated that IgG accumulation rather than IgA accumulation in mesangium was associated with poor renal outcome [1, 4, 5]. Animal models showed that the increased level of glomerular IgG accumulation is linked to the increased level of proteinuria [6]. Therefore, renal immunofluorescence microscopy (IFM) could also provide clinical or prognostic information. IFM fails to show IgG in up to 50–80% of kidney biopsies [1]; however, using more powerful methods, e.g. confocal microscopy, could help show glomerular IgG deposits. Thus, albeit not intensely stained or not detected with IFM, glomerular IgG may be associated with poor renal outcomes, such as severe staining cases.

The aim of this study is to determine whether IgG positivity and intensity of IgG staining detected by routine immunofluorescence microscopy (IFM) is associated with poor renal prognostic criteria.

## Methods

### Study design and subjects

This study is a retrospective, multicentre, cross-sectional study between May 2009–May 2019. The data of patients who underwent native kidney biopsy and diagnosed with ‘primary glomerular disease’ extracted from the ‘Primary Glomerulonephritis Registry of Turkish Society of Nephrology’ database by TSN-GOLD Working Group. Between 2009 and 2019, a total of 4399 patients from 47 nephrology centres all over Turkey were evaluated. Patients aged 16 years or more with documented biopsy findings were included in the study. After excluding patients with missing kidney biopsy findings, the data of 3875 patients were evaluated. Among 3875 patients, a total of 943 patients had primary IgAN. The present study was approved by the Ethical Committee of the Istanbul Medical Faculty of Istanbul University.

#### Renal histopathology

The specimens were evaluated by the procedure under light microscopy and IFM. Light microscopic examination was carried out with paraffin-hidden tissue under hematoxylin-eosin, periodic acid-Schiff, aldehyde fuchsin orange G, and periodic acid-silver methenamine stains. Snap-frozen 3-μm-thick sections were used for routine IFM. Snap-frozen 3-μm-thick sections were encountered with IgG antibodies beforehand. The semiquantitative force of IgG deposits in the mesangium, or glomerular capillary loops were evaluated by IFM in order to detect the presence of glomerular IgG. The deficiency of deposits in the mesangium or glomerular capillary loops were considered IgG negative. The presence of deposits in the mesangium or glomerular capillary loops were considered IgG positive. The IgG accumulation was scored as mild [IgG (+)], moderate [IgG (++)] and severe [IgG (+++)].

The total number of glomeruli, global sclerotic glomeruli, and segmental sclerotic crescentic glomeruli were evaluated in the renal biopsies’ materials. In addition, thickening of the basal membrane, mesangial proliferation, endocapillary proliferation and interstitial inflammation was evaluated. In addition, histopathologic findings such as mesangial hypercellularity, endocapillary hypercellularity, segmental sclerosis, tubular atrophy/interstitial fibrosis belonging in the Oxford classification were also evaluated. The mesangial cellularity was scored as 0 (< 4 mesangial cells/mesangial area), 1 (4–5 cells), 2 (6–7 cells), and 3 (≥8 cells) for each glomerulus. The mean score of all glomeruli was classified as M0 (≤0.5) and M1 (> 0.5). Endocapillary hypercellularity was categorised as either present (E1) or absent (E0). Similarly, segmental glomerulosclerosis was classified as either present (S1) or absent (S0). Tubular atrophy/interstitial fibrosis was classified as T0 (0–25% of the cortical area), T1 (26–50% of the cortical area), or T2 (> 50% of the cortical area) [7]. Interstitial fibrosis was defined as an increased extracellular matrix separating tubules in the cortical area. It is scored as percentage involvement, where < 1% was denoted as the absence of interstitial fibrosis (stage F0), with 1–5% rounded to 5% (stage F1, moderate interstitial fibrosis) and other values rounded to the nearest 10% (stage F2, severe interstitial fibrosis) [8].

#### Data collection

Age, gender, BMI, blood pressure and laboratory parameters including complete blood count, lipid profile, renal function tests, albumin, total protein, glucose, uric acid, calcium, alanine aminotransferase (ALT), complement 3 (C3), and complement 4 (C4) levels, 24-h proteinuria were collected and evaluated. Statistical differences of these parameters were evaluated between glomerular IgG negative and positive patients. These parameters were also evaluated in the subgroup analysis performed in IgG positive patients. The correlation between the intensity of glomerular IgG positive staining and age, urea, creatinine, eGFR, uric acid, albumin, urinary proteinuria, systolic blood pressure, diastolic blood pressure was evaluated.

The average of at least 2 systolic and diastolic blood pressure measurements was used. 24-h urine was collected to determine 24-h protein excretion. It was performed twice, and the average was calculated. eGFR was calculated by CKD-EPI formula [Chronic Kidney Disease Epidemiology Collaboration) = 141 X min (Scr/κ,1)α X max (Scr/κ,1)-1.209 X 0.993age × 1.018 [female] X 1.159 [black]) [9]. Low-density lipoprotein cholesterol (LDL-C) level was assessed with Friedewald formula, which can be formulated as LDL = TC − (HDL) − (TG / 5) [10].

#### Statistical analysis

Statistical analyses were performed using Statistical Package for the Social Sciences (SPSS 22) software. Numerical variables that exhibited normal distribution were given as mean ± standard deviation; the categorical variables as frequency and percentage. In the comparative analysis of demographic, laboratory and histopathological characteristics between IgG positive staining patients and IgG negative staining patients (Tables 1 and 3), independent groups were performed using the t-test for parameters with normal distribution and the Chi-square test was used in the comparisons between categorical variables. ANOVA test was used to compare three groups according to the IgG staining intensity (Tables 2 and 4). For all statistical analyses, *p*-value ≤0.05 was considered to be statistically significant.

**Table 1:** Basic demographic and biochemical findings of IgG positive and IgG negative patients

	IgG positive patients (n: 179)	IgG negative patients (n: 764)	p
**Age (years)**	38.5 ± 13.1	38.4 ± 12.8	NS
**Gender (male)**	63.7%	62.7%	NS
**BMI (kg/m**^**2**^**)**	26.4 ± 5.1	26.7 ± 5.1	NS
**SBP (mmHg)**	130 ± 22	130 ± 20	NS
**DBP (mmHg)**	82 ± 13	81 ± 12	NS
**FBG (mg/dl)**	97 ± 29	95 ± 23	NS
**BUN (mg/dl)**	26 ± 22	24 ± 17	NS
**Creatinine (mg/dl)**	1.51 ± 1.36	1.56 ± 1.25	NS
**eGFR (ml/min/1.73 m**^**2**^**)**	69.7 ± 37.9	66.5 ± 36.8	NS
**Proteinuria (g/day)**	3.20 ± 0.48	3.24 ± 0.45	NS
**Albumin (g/dl)**	3.9 ± 0.6	3.8 ± 0.7	NS
**Uric acid (mg/dl)**	6.6 ± 1.9	6.6 ± 2.0	NS
**Total protein (g/dl)**	7.6 ± 0.9	7.6 ± 0.9	NS
**Total cholesterol (mg/dl)**	206 ± 49	215 ± 62	NS
**LDL (mg/dl)**	129 ± 41	133 ± 50	NS
**HDL (mg/dl)**	46 ± 17	46 ± 16	NS
Triglycerides (mg/dl)*	163 ± 99	187 ± 123	< 0.05
**Hemoglobin (g/dl)**	13 ± 2	13 ± 2	NS
**Pyuria (leukocyte > 5)**	17.7%	20.6%	NS
**Haematuria (erythrocyte > 5)**	70%	72%	NS
**C3 low**	6.6%	8.4%	NS
**C4 low**	0.9%	0.9%	NS

*Abbreviations*: *BMI* Body mass index, *SBP* Systolic blood pressure, *DBP* Diastolic blood pressure, *FBG* fasting blood glucose, *BUN* Blood urea nitrogen, *eGFR* estimated glomerular filtration rate, *LDL* Low density lipoprotein, *HDL* High density lipoprotein, *C* Complement

**Table 2:** Basic demographic and biochemical analyses of IgG positive patient subgroup

	IgG (+)	IgG (++)	IgG (+++)	p
	(n: 131)	(n: 36)	(n: 12)	
**Age (years)**	38.2 ± 12.8	40.3 ± 15.0	36.5 ± 11.2	NS
**Gender (male)**	63.4%	63.9%	66.7%	NS
**BMI (kg/m**^**2**^**)**	26.4 ± 5.1	26.1 ± 4.6	27.5 ± 10.4	NS
**SBP (mmHg)**	131 ± 23	128 ± 22	119 ± 15	NS
**DBP (mmHg)**	83 ± 12	80 ± 14	81 ± 13	NS
**FBG(mg/dl)**	99 ± 31	93 ± 22	90 ± 21	NS
**BUN (mg/dl)**	26 ± 23	26 ± 20	24 ± 12	NS
**Creatinine (mg/dl)**	1.56 ± 1.53	1.39 ± 0.69	1:32 ± 0.34	NS
**eGFR (ml/min/1.73 m**^**2**^**)**	70.4 ± 39.0	69.6 ± 38.7	61.6 ± 16.7	NS
**Proteinuria (g/day)**	3.16 ± 0.50	3.27 ± 0.38	3.37 ± 0.48	NS
**Albumin (g/dl)**	3.9 ± 0.5	3.9 ± 0.9	3.6 ± 0.7	NS
**Uric acid (mg/dl)**	6.6 ± 2.0	7.6 ± 1.8	7.1 ± 1.5	NS
**C3 low**	7.5%	8%	10%	NS
**C4 low**	5.5%	5.2%	6%	NS
**Haematuria (erythrocytes > 5)**	69.7%	75%	87.5%	NS

*Abbreviations*: *BMI* Body mass index, *SBP* Systolic blood pressure, *DBP* Diastolic blood pressure, *FBG* fasting blood glucose, *BUN* Blood urea nitrogen, *eGFR* estimated glomerular filtration rate, *C* Complement

## Results

81% (*n* = 764) of the patients were detected as IgG negative, while 19% (*n* = 179) were positive with IFM. However, subgroup analysis of glomerular IgG positive patients under IFM showed as follows: IgG (+) *n* = 131, IgG (++) *n* = 36, IgG (+++) *n* = 12.

### Basic demographic and biochemical analyses of IgG negative and positive patients during the biopsy

63.7% of glomerular IgG positive patients and 62.7% of IgG negative patients were male (*p* > 0.05). While the mean eGFR of glomerular IgG positive patients was 69.7 ± 37.9, it was 66.5 ± 36.8 ml/min/1.73 m^2^ for IgG negative patients (p > 0.05). The mean value of 24-h urinary proteinuria was 3.20 ± 0.48 g for glomerular IgG positive patients, whereas it was 3.24 ± 0.45 g for IgG negative patients (p > 0.05).

Demographic and biochemical findings of glomerular IgG positive and negative patients during the biopsy were shown in Table 1.

Intensity of glomerular IgG positivity was not associated with diastolic blood pressure, systolic blood pressure, urea, uric acid, age, eGFR, albumin, creatinine, 24-h urinary proteinuria or low serum C3 and C4 (for all p > 0.05, r = − 0.084, r = − 0.102, r = − 0.006, r = 0.062, r = 0.014, r = − 0.044, r = − 0.061, r = − 0.066, r = 0.150, r = − 0.103, r = 0.012, respectively).

### Basic demographic and biochemical analyses according to the intensity of glomerular IgG staining

Basic demographic and biochemical analyses of the glomerular IgG positive patients were shown in Table 2. There was no statistically significant difference between the groups in terms of the number of male patients, systolic blood pressure, diastolic blood pressure, BMI, uric acid, BUN, Creatinine, albumin, 24-h proteinuria or age.

### Histopathologic findings of IgG negative and positive patients during the biopsy

Although endocapillary hypercellularity was significantly higher in patients with glomerular IgG positive compared to IgG negatives, there was no statistical difference (*p* > 0.05). T1 score of glomerular IgG negative patients was significantly higher compared to IgG positive patients (*p* < 0.05). Histopathologic findings and statistically significant differences between glomerular IgG positive and negative patients were shown in Table 3.

**Table 3:** Histopathologic findings of IgG positive and IgG negative patients

	IgG positive patients (n: 179)	IgG negative patients (n: 764)	p
**Total glomerulus number**	18 ± 10	17 ± 10	NS
**Global sclerotic glomerulus number**	4 ± 4	4 ± 4	NS
**Segmental sclerotic glomerulus number**	2 ± 3	1 ± 2	NS
**Cellular crescent number**	0 ± 1	0 ± 1	NS
**Fibrocellular crescent number**	0 ± 1	0 ± 2	NS
**Basal membrane thickening**	22.9%	20.9%	NS
**Mesangial proliferation**	86.2%	86.4%	NS
**Endocapillary proliferation**	24.2%	18.3%	NS
**Interstitial inflammation**	72.0%	74.7%	NS
**Tubular atrophy/interstitial fibrosis**			
**T0**	58.5%	41.3%	< 0.05
**T1**	35.3%	44.7%	< 0.05
**T2**	6.2%	11%	NS
**IgA staining**			
**(+)**	5.6%	5.9%	NS
**(++)**	27.5%	33.5%	NS
**(+++)**	68.6%	60.5%	NS
**Mesangial hypercellularity**			
**M0**	23.5%	22.2%	NS
**M1**	76.5%	78.8%	NS
**Endocapillary hypercellularity**			
**E0**	66.4%	78.7%	NS
**E1**	33.6%	21.3%	NS
**Segmental glomerulosclerosis**			
**S0**	44.9%	39.5%	NS
**S1**	55.1%	60.5%	NS

### Histopathologic findings according to the intensity of glomerular IgG staining during the biopsy

There was no difference for histopathological findings between IgG (+), IgG (++), IgG (+++) groups (for all, p > 0.05). Histopathologic findings of the glomerular IgG positive patients’ and their comparative analyses were shown in Table 4.

**Table 4:** Histopathologic results of IgG positive patient subgroup

	IgG (+)	IgG (++)	IgG (+++)	p
**Total glomerulus number**	18 ± 10	18 ± 12	22 ± 10	NS
**Global sclerotic glomerulus number**	4 ± 4	4 ± 5	5 ± 3	NS
**Segmental sclerotic glomerulus number**	2 ± 3	2 ± 3	2 ± 2	NS
**Cellular crescent number**	1 ± 2	1 ± 2	2 ± 4	NS
**Basal membrane thickening**	25.8%	17.6%	25.0%	NS
**Mesangial proliferation**	86.7%	82.8%	91.6%	NS
**Endocapillary proliferation**	22.8%	24.1%	42.8%	NS
**Interstitial inflammation**	71.3%	71.4%	81.8%	NS
**Tubular atrophy/interstitial fibrosis**				
**T0**	62.5%	52.8%	50.0%	NS
**T1**	30.5%	42.8%	33.3%	NS
**T2**	7%	4.9%	66.7%	NS
**IgA staining**				
**(+)**	6.1%	0%	19.1%	NS
**(++)**	28.7%	27.7%	9.2%	NS
**(+++)**	65.2%	62.3%	72.7%	NS
**Mesangial hypercellularity**				
**M0**	26.7%	14.2%	16.6%	NS
**M1**	73%	85.8%	%83.4%	NS
**Endocapillary hypercellularity**				
**E0**	66.1	71.4	50.0%	NS
**E1**	33.9%	28.6%	50.0%	NS
**Segmental glomerulosclerosis**				
**S0**	%45.0%	38.0	66.6	NS
**S1**	%55.0%	62.0	33.4	NS

## Discussion

Several studies described the relationship between glomerular IgG staining and poor prognostic markers in patients with IgA nephropathy. The difference between our study from these studies is that this study is a multi-centre study with a large number of patients. To the best of our knowledge, our study is the first study that the intensity of glomerular IgG staining in IgAN patients was not related to poor prognostic factors and emphasises the importance of IgG positivity, as well as IgG negativity in IgAN patients.

In this retrospective study performed by the TSN-GOLD Working Group, no difference was found between glomerular IgG negative and IgG positive IgAN patients for Oxford scores, biochemical, histopathological findings. In addition, no association between the intensity of glomerular IgG staining and poor renal prognostic factors such as 24-h urinary proteinuria, eGFR, creatinine, albumin, age, uric acid, systolic and diastolic blood pressures were found. Although the presence of glomerular in situ IgG/Gd-IgA1 immunocomplexes was shown in IgAN [11, 12], some experts suggest that routine IFM may be unsuccessful in detecting actually existing glomerular IgG [13, 14]. There is some evidence that both deposition of IgG in glomerular mesangial space and in glomerular capillary loops are associated with poor prognostic factors. Although it has been reported that IgG accumulation in both mesangium and capillary loops is not different for poor renal prognostic factor [14], some studies have reported that IgG accumulation in capillary loops is highly related to poor renal prognosis than mesangial IgG accumulation [5]. As a result, glomerular IgG deposits in IgAN, independent from the location, may be associated with poor renal prognosis. In our study, we considered IgG positive if the presence of IgG deposits in the mesangium or glomerular capillary loops were positive. We did not find any difference between poor renal prognostic factors of patients with IgG negative and IgG positive. Moreover, we could not find a relationship between the intensity of the IgG positivity and poor renal prognostic factors in patients with IgG positive.

Galactose deficient IgA1 is formed in the circulation due to O-glycosylation abnormality in the hinge region of IgA1s in patients with IgAN. Lymphocytes produce IgG against abnormal IgA1. IgG/Gd-IgA1 in situ immunocomplexes are formed, and these complexes accumulate in the mesangial or glomerular capillary loops in the glomeruli [15, 16]. It is known that these in situ immunocomplexes are locally activating alternative and mannose-binding lectin pathways of the complement in IgAN [17]. IgG/Gd-IgA1 in situ immune complexes are cause the formation of the C4bC2a classical pathway by C1 fixation. As a result, C3 is converting to C3a and C3b. Activation of complements is causing the formation of C3 convertase in the mesangium. C3 convertase cause C3 fragment and fragmented products of C3 [6].

According to this theory, C3 deposits in the mesangium causes severe histological lesions such as glomerulosclerosis or crescent formation. Moreover, the presence of IgG in the mesangial cells causes mesangiolysis and mesangial cellular activation in IgAN [18]. Shin et al. observed in their study that there was a positive correlation between IgG and C3 deposition, and glomerular IgG was associated with tubulointerstitial fibrosis in IgAN [4]. In our study, there was no correlation between glomerular IgG and glomerular C3 deposition. Chen et al. informed that low serum C3 levels were associated with poor prognostic factors in IgAN [15]. In our study, there was no correlation between low serum C3 levels and glomerular IgG positivity. Also, there was no difference in serum C3 level between IgG positive and negative patients.

IgA positivity alone is observed at a rate of approximately 25% by IFM in patients with IgAN [19]. On the other hand, IgG positivity has been occurred at a rate of 10–80% in IgAN [20]. IgG positivity in IgAN could aggravate glomerular inflammation and proteinuria [6]. It was reported in a Japanese cohort study that deposition of IgG in the capillary loops were increased proteinuria and associated with decreased renal functions [21]. In another study, it was reported that mesangial IgG deposition is associated with hypertension and decreased renal functions in IgAN patients [22]. A Korean study emphasised that the presence of glomerular IgG in IgAN patients is an independent risk factor for a poor renal outcome [4]. In a Japanese cohort study was reported that the intensity of IgG in the capillary loops was associated with a decrease in eGFR [23]. Likewise, IgG deposition in the mesangium and the capillary loops were associated with mortality and risk of renal replacement therapy in an Italian study [24]. However, in our study, there was no difference between IgG negative and IgG positive patients regarding poor prognostic criteria such as the amount of proteinuria, blood pressures, serum creatinine and eGFR levels. Moreover, no correlation was found between the intensity of IgG staining and the amount of proteinuria, serum creatinine, eGFR, or blood pressures. It is known that there is an association between Oxford classification and clinical renal outcome in IgAN [8]. However, glomerular IgG positivity was not evaluated in the Oxford classification. There are several studies in IgAN showing the relationship between IgG positivity detected by IFM and histological severity. Although Bellur et al. had shown the relationship between glomerular IgG positivity and endocapillary proliferation as well as mesangial cellularity, they could not find an association between IgG positivity and worse renal outcome [14]. In our study, there was no difference in Oxford scores of IgG positivity patients compared to IgG negative patients. In addition, as the intensity of IgG positivity increased, there was no difference in Oxford scores.

Rizk et al. examined kidney biopsies of IgG negative and positive in IgAN patients. They examined a total of 34 IgAN patients who were 14 IgG positive and 24 IgG negative with IFM. They included 6 primary membranous nephropathy and 8 lupus nephritis patients in their study as a control group [1]. IgG’s were extracted from the renal biopsy materials of all the patients, and they observed the number of IgG’s by ELISA. Confocal microscopy was used in six IgG positives and four IgG negative patients under IFM. Rizk et al. determined that the IgG autoantibodies in the kidney biopsy immunodeposits which were detected by both ELISA and IgG-IgA1 antigen were specific for Gd-IgA1. Even IgG negative by IFM, these autoantibodies were obtained when using either ELISA or IgG-IgA1 antigen by Rizk et al. In IgG negative biopsy samples, which were detected by using routine IFM, Rizk et al. demonstrated glomerular colocalisation of IgG and IgA using by confocal microscopy. These suggest that IgG positive biopsy samples have more Gd-IgA1-IgG immunocomplexes compared to IgG negatives.

Renal immune deposits in IgAN may be rich in IgG autoantibodies which are specific for Gd-IgA1, even if IgG’s are not detected by routine IFM in IgAN kidney-biopsy specimens [1]. For these reasons, we may not have found a difference of poor renal prognostic risk factors between IgG negative and IgG positive patients in our study. Additionally, in our study, this may be one of the reasons why IgG positivity was not associated with poor renal prognostic risk factors. Even if IgG detected by routine IFM in IgAN glomeruli is mild, IgG may actually be severely positive.

### Limitations

There are some limitations in our study: First, we didn’t have follow-up data; therefore, we could not evaluate the association of IgG deposition with renal outcomes. Second, biopsy specimens were evaluated by different pathologists in each centre. Finally, IgG evaluation was not performed using confocal microscopy.

## Conclusions

Glomerular IgG positivity detected by routine IFM in IgAN is not found to be associated with poor renal prognostic markers. Glomerular IgG negative and IgG positivity detected by routine IFM in IgAN patients is not associated with poor renal prognostic risk factors. Therefore, IgG evaluated by routine IFM might not be suitable for existing IgG severity detection.

## Data Availability

The datasets used and/or analysed during the current study available from the corresponding author on reasonable request.

## Abbreviations

BMI: body mass index
IgAN: IgA nephropathy
C: Complement
CKD-EPI Formula: Chronic Kidney Disease Epidemiology Collaboration
eGFR: Estimated glomerular filtration rate
Gd-IgA1: Galactose deficient IgA1
IFM: Immunofluorescent microscopy
LM: Light microscopy
LDL-C: Low-density lipoprotein cholesterol
